# Phenotypic and genotypic identification of carbapenem resistance in *Bacteroides fragilis* clinical strains

**DOI:** 10.1007/s00430-023-00765-w

**Published:** 2023-05-13

**Authors:** Marta Kierzkowska, Anna Majewska, Konrad Karłowicz, Hanna Pituch

**Affiliations:** Department of Medical Microbiology, Medical University of Warsaw, Warsaw, Poland

**Keywords:** *Bacteroides fragilis*, Carba NP, Carbapenemase, *Cfi*A, Meropenem

## Abstract

*Bacteroides fragilis* is an important etiological agent of serious infections in humans. Rapid methods, readily adaptable to use in medical laboratories, are needed to detect antibiotic resistance and decrease the likelihood of therapy failure. The aim of this study was to determine the prevalence of *B. fragilis cfi*A-positive isolates. The second purpose was to investigate the carbapenemase activity in *B. fragilis* strains by Carba NP test. In the study, 5.2% of *B. fragilis* isolates are phenotypically resistant to meropenem. The *cfiA* gene was identified in 6.1% of *B. fragilis* isolates. The MICs of meropenem were significantly higher in *cfi*A-positive strains. The presence of the *cfi*A gene along with the IS*1186* was detected in one *B. fragilis* strain which was resistant to meropenem (MIC 1.5 mg/L). The Carba NP test results were positive for all the *cfi*A-positive strains, including those susceptible to carbapenems based on their MIC values. A review of the literature revealed that the rate of *B. fragilis* with the *cfi*A gene varies from 7.6 to 38.9% worldwide. Presented results are in line with the other European studies. Phenotypic testing with the Carba NP test, it seems to be a viable alternative for the *cfi*A gene detection in *B. fragilis* isolates. The positive result obtained is of greater clinical importance than the detection of the gene *cfi*A.

**Supplementary Information:**

The online version contains supplementary material available at 10.1007/s00430-023-00765-w.

## Introduction

Members of the genus *Bacteroides* are a component of the human microbiota. They colonize the gastrointestinal tract, distal part of the genitourinary system, and the upper airways. Many species are opportunistic pathogens, responsible for endogenous infections [1, 2, 3].

*Bacteroides fragilis* is considered the most important species, with infection rates of 60–80% and is the most frequently identified anaerobic bacteria (excluding *Clostridioides difficile* recovered from patients with antibiotic diarrhea) in the clinical laboratories. It is isolated from mono- and polymicrobial infections. It occurs in specimens taken from sites of infection following violation of natural barriers by surgery, inflammation, or trauma. Intra-abdominal infections are the most common form of infection [1, 4].

Laboratory diagnostics of anaerobes is one of the most challenging aspects of clinical bacteriology. *Bacteroides* sp. isolation, identification, and antibiotic susceptibility testing (AST) is time consuming and labor intensive. Thus, anaerobes can be omitted from the routine diagnostic in many medical laboratories [5, 6, 7, 8, 9].

Infections caused by *B. fragilis* should be treated according to the results of AST because of an increasing resistance to commonly used antibiotics including β-lactams, tetracyclines, macrolides, and fluoroquinolones [8]. Carbapenems are among the most effective drugs and are considered the drug of choice for therapy of complicated intra-abdominal infections, acute gynecological infections, and skin and soft tissue infections caused by *B. fragilis.* Such infections are often polybacterial and also caused by other Gram-negative bacilli [10, 11].

Clinical isolates of *B. fragilis* may be resistant to carbapenems, considered to be the last chance β-lactam antibiotic, so resistance should be monitored extensively [12, 13, 14, 15, 16].

Resistance to carbapenems in *B. fragilis* is usually caused by the expression of the class B metallo-beta-lactamase encoded by, located on the chromosome *cfiA* gene. *cfi*A encodes an Ambler class B zinc metallo-β-lactamase (MBL) that can hydrolyzes most of β-lactams, including cephamycins and carbapenems [10, 17]. On this basis, *Bacteroides* spp. can be classified into Division I (*cfi*A-negative) and Division II (*cfi*A-positive) [17]. Anaerobes that produce MBL enzyme are the most worrisome. They hydrolyze nearly all β-lactam antibiotics, except monobactam [10, 18, 19]. These β-lactamase are not inactivated by currently known β-lactamase inhibitors [18]. *cfi*A gene is considered as a silent gene with a low level of constitutive expression. Its expression can be upregulated following the insertion of an insertion sequence (IS) with an efficient promotor immediately upstream of the gene [17, 20].

Rapid methods, readily adaptable and optimized for use in medical laboratories, are needed to detect antibiotic resistance in anaerobic bacteria and decrease the likelihood of carbapenem therapy failure [21].

Detection of *cfi*A gene is not an optimal method for routine identification of strains resistant to carbapenems. Proteins cannot be expressed at a sufficiently high level to classify the strains as resistant [18, 22]. Phenotypic imipenem-EDTA double disk synergy test for the detection of metallo‐β‐lactamases produced by Gram-negative aerobic bacilli, has no or restricted application with anaerobic bacteria. *B. fragilis* isolates with the *cfi*A gene can be susceptible or intermediate to imipenem and have a negative imipenem double-ended E-test result but be resistant to meropenem [23, 24]. Double-ended E-test strips impregnated with meropenem or imipenem with or without EDTA has been proposed as well. A preliminary analysis indicated that sensitivity is highly variable and depends on the carbapenem used and the resistance level of the strains tested [21, 24].

A potentially applicable for routine use method identifying carbapenemase production is a biochemical method relying on imipenem hydrolysis—the Carba NP test, originally intended for the aerobic *Enterobacteriaceae* bacilli [25, 26]. There are several reports concerning the application of Carba NP test for anaerobic bacteria [27, 28, 29]. The potential use of this method should be based on evidence resulting from studies with clinical strains performed under conditions simulating routine work in a clinical microbiology laboratory.

### Aim

The aim of this study was to determine the prevalence of *B. fragilis cfiA*-positive (Division II) isolates to assess its influence on phenotypic resistance to carbapenems. The second purpose was to investigate the carbapenemase activity in *B. fragilis* strains by Carba NP test and to compare the outcome with the phenotypic (MICs evaluation) and genotypic (*cfi*A and IS genes detection) test results.

## Materials and methods

### Bacterial strains

The study was performed at the microbiology laboratory that served bacteriological samples from a major academic hospital in Warsaw, Poland, the Medical University of Warsaw. Altogether, 115 consecutive non-duplicate *B. fragilis* isolates were analyzed over a period of 5 years between January 2013 and December 2017. Strains were cultured from the following clinical specimens: wound/abscess swabs (81), peritoneal cavity fluid (14), blood (6), soft tissue (5), others (9).

Clinical sample was plated on Schaedler agar media with 5% sheep blood, vitamin K, and hemin (bioMérieux, France) and was incubated at 37 °C in an anaerobic atmosphere (anaerostat Genbox System providing air composition: 85% N_2_, 10% H_2_, and 5% CO_2_; bioMérieux). Incubation period lasted 24–48 h. Bacterial identification was carried out using the matrix-assisted laser desorption ionization time-of-flight mass spectrometry (MALDI-TOF MS, bioMérieux). All the strains were stored deep-frozen, in a temperature of − 70 °C, in bead vials Protect Select (Technical Service Consultants Ltd, UK). To perform phenotypic and molecular tests, the strains were revived by culturing on Schaedler agar.

### Antibiotic susceptibility test and interpretation

The E-test strips impregnated with a concentration gradient of imipenem (0.002–32 mg/L) and meropenem (0.002–32 mg/L) were used for detection of a minimum inhibitory concentration (MIC) of carbapenems. E-test assays were performed as recommended by the manufacturer (bioMérieux, France). The interpretation was conducted in accordance with The European Committee on Antimicrobial Susceptibility Testing (EUCAST; version 12.0 which complies with version 13; year 2023) recommendations and according to results of Rennie et al. on the assessment of drug susceptibility of anaerobic bacteria [30, 31]. MIC_90_ and MIC_50_ values were defined as the lowest concentration of the antibiotic at which 90 and 50% of the isolates were inhibited, respectively. The strain from the American Type Culture Collection: *Bacteroides fragilis* ATCC 25285 was used as control. In 2022, EUCAST changed the interpretations of antibiotic susceptibility for *Bacteroides* spp. [30, 32]. The clinical MIC breakpoints for meropenem have been changed so that MIC breakpoint > 1 mg/L was interpreted as resistant to meropenem. According to the earlier version of EUCAST (v. 11.0 from 2021), a MIC of > 8 mg/L of meropenem indicated resistance to this antibiotic. Interpretation for imipenem has been withdrawn [30, 32].

### The Carba NP test

The Carba NP (Carbapenemase Nordmann–Poirel) test is a phenotypic method that was developed to detect carbapenemase produced by Gram-negative aerobic bacteria, including *Enterobacteriaceae* and *Pseudomonas* *aeruginosa* isolates [33, 34]. A variant of the test, the CarbAcineto, allows for the detection of acquired carbapenemases in *Acinetobacter* spp. [25].

The Carba NP test is based on In vitro detection of hydrolysis of imipenem by a bacterial lysate suspended in a buffer containing phenol red. As a result of imipenem hydrolysis, the pH of the reaction medium decreases (acidification), which is observed as a change in the color of phenol red to yellow or orange. A positive result indicates carbapenemase production by the strain. Positive control (carbapenemase-producing isolate) and negative control (carbapenemase-not-producing isolate) were included in the study to assess the correctness of the test performed.

The Carba NP test was performed as follows: one loopful of bacteria, approximately 10 μL (incubated for 48 h at 30 ºC on Schaedler agar) was resuspended in a Tris–HCl 20 mmol/L lysis buffer (B-PERII, Bacterial Protein Extraction Reagent; Thermo Scientific Pierce, Rockford, IL, USA), vortexed for 1 min. and further incubated at room temperature for 30 min., then centrifuged at 10,000 × g at room temperature for 5 min. In the test tube, the supernatant was mixed with 100 µL of a 1 mL solution made of 3 mg of imipenem monohydrate (pH 7.8), phenol red solution (both Sigma, Saint-Quentin Fallavier, France) and 0.1 mmol/L ZnSO4 (Merck Millipore, Guyancourt, France). In the control tube, the supernatant was mixed with the phenol red solution (prepared by mixing 2 mL of a phenol red solution 0.5% (wt/vol) with 16.6 mL of distilled water). The pH value was then adjusted to using a pH meter to 7.8 by adding drops of 1 N NaOH.

A mixture was incubated at 37 °C for a maximum of 2 h. The test was read by comparing the color of the mixture in the test and the control tubes. When imipenem was hydrolyzed, the colur has been turned from red to orange or yellow, which was interpreted as a positive Carba NP test. Tubes containing bacterial extracts with no carbapenemase activity remained red (negative). In the case of a slight colour change, the result was considered invalid, and the test was repeated. A carbapenemase-producing strain BF8 *B. fragilis* (BFr81) was used as the positive control, and *B. fragilis* ATCC 25285 strain as the negative control [28, 34].

### The *cfi*A-mediated carbapenem resistance gene and insertion sequence-encoding genes detection

*cfi*A gene was detected by polymerase chain reaction (PCR) using specific primer pairs [35]. The isolates identified as *cfi*A-positive were also evaluated for insertion sequence-encoding genes (IS*1186*, IS*1187*, IS*1188*, IS*942*) [36, 37]. The DNA was collected using a genomic DNA isolation kit for bacteria, cell cultures, and solid tissue (Genomic Mini; A&A Biotechnology, Poland). The starters were ordered and synthesized at the Laboratory of DNA Sequencing and Oligonucleotide Synthesis at the Institute of Biochemistry and Biophysics Polish Academy of Sciences (Warsaw, Poland). The obtained DNA fragments were subsequently separated using electrophoresis in 1% agarose gel with ethidium bromide to identify PCR products and then observed in a gel imaging device. BF8 *B. fragilis* (BFr81) was used as the positive control in the PCR test. PCR primers and conditions are listed in Table 1.

**Table 1:** PCR primers and reaction conditions for the detection of *cfi*A and IS genes [35, 36, 37]

Genes	5′ → 3′ primer	PCR					
		Initial denaturation	Denaturation	Annealing		Extension	Final extension
			Cycle count				
*cfi*A gene							
* cfi*A	CCATGCTTTTCCCTGTCGCAG	95 ºC 5 min	95 °C 1 min	51 °C 30 s		72 °C 40 s	72 °C 7 min
	GGGCTATGGCTTTGAAGTGC		35x				
IS gene							
IS*1186*	GAGAATCAAGCTTCTCGCC	95 °C	95 °C 30 s	57 °C 30 s		72 °C 1,5 min	72 °C
	CCCCGAATTCGCCTTTGCCCGTA	5 min	35 x				5 min
IS*1187*	CGTATTGCAGAATGGTAAGTGC	95 °C	95 °C 30 s	54 °C 30 s		72 °C 1 min	72 °C
	GTTCCACGTCGTGGTCCTGTTC	5 min	35 x				5 min
IS*1188*	GGCCTGTGCTCACAACCGAC	95 °C	95 °C 30 s	55 °C 30 s		72 °C 1 min	72 °C
	CGGTATGCGGTCACATATGC	5 min	35 x				5 min
IS*942*	TCTGAGAAACTCACTCCTTTTGGAGGA	95 °C	95 °C 30 s	55 °C 30 s		72 °C 1,5 min	72 °C
	AGAAAAGCATGGTCTTTAACCAAAGTC	5 min	35 x				5 min

## Statistical analysis

Statistical analysis was conducted with Statistica 10 (StatSoft, Inc.). Any correlations between the presence of resistant gene (*cfi*A) in the evaluated isolates and antibiotic MIC values were analyzed with linear regression using the Pearson method. The obtained correlation coefficients (r) were interpreted as follows: *r* = 0, no correlation; 0 < r ≤ 0.1, very weak correlation; 0.1 < r ≤ 0.3, weak correlation; 0.3 < r ≤ 0.5, moderate correlation; 0.5 < r ≤ 0.7, strong correlation; 0.7 < r ≤ 0.9, very strong correlation; 0.9 < r < 1, almost perfect correlation; and *r* = 1, perfect correlation. The *p* value was calculated for each correlation coefficient and was considered statistically significant at *p* ≤ 0.05.

## Results

Using the currently applicable criteria for interpreting phenotypic antibiotic susceptibility tests according to the recommendations of EUCAST, it has been shown that 5.2% (6/115) of *B. fragilis* strains are resistant to meropenem. Assuming earlier (version 11.0; EUCAST, 2021) breakpoint values [32], only two (1.73%) strains resistant to meropenem and one (0.87%) intermediate could be identified in the tested pool of clinical strains (Table 2). According to the up-to-date recommendations, isolates with an MIC of 1 mg/L may harbor the *cfi*A gene. Table 2. Characterizes isolates that are phenotypically resistant to any of the carbapenems and/or had detected sequences that may be associated with drug resistance to these antibiotics. Imipenem MIC_50_ and MIC_90_ were 0.125 and 0.87 mg/L, respectively. Meropenem MIC_50_ and MIC_90_ were 0.094 and 0.25 mg/L, respectively.

**Table 2:** Characterization of isolates phenotypically resistant to imipenem and meropenem and/or with *cfi*A, IS, carbapenemase activity detection

N	ID	Clinical sample	Hospital ward	MIC IP [mg/L]	Interpretation	MIC MP[mg/L]	Interpretation		Carba NP test	*cfi*A	IS 1186	MIC MZ [mg/L]			MIC CM [mg/L]	
					EUCAST 2021		EUCAST 2021	*EUCAST 2022								
1	12	Wound/abscess	Gynecology	0.25	S	12	R	R	+	+	−	0.125	S		4	S
2	66	Wound/abscess	General surgery	32	R	32	R	R	+	+	−	0.125	S		2	S
3	76	Wound/abscess	Dermatology	0.047	S	0.5	S	S	+	+	−	0.032	S		0.016	S
4	82	Intraperitoneal fluid	General surgery	0.125	S	1	S	S	+	+	−	0.032	S		0.75	S
5	87	Soft tissue	Urology	0.125	S	1.5	S	R	+	+	+	0.25	S		3	S
6	88	Wound/abscess	General surgery	0.19	S	2	S	R	+	+	−	0.094	S		0.75	S
7	115	Wound/abscess	General surgery	0.125	S	2	S	R	+	+	−	0.25	S		0,125	S
8	221	Wound/abscess	General surgery	0.064	S	4	I	R	−	−	−	0.023	S		0.047	S

*Changes compared to the previous version. EUCAST version 12 (year 2022) complies with version 13 (year 2023)

According to the recommendations, version 12 (2022) some isolates with an MIC of 1 mg/L may harbor the *cfi*A gene

*IP* imipenem, *MP* meropenem, *MZ* metronidazole, *CM* clindamycin

S susceptible, a microorganism is categorized as susceptible when there is a high likelihood of therapeutic success using a standard dosing regimen of the agent

I susceptible, a microorganism is categorized as susceptible, when there is a high likelihood of therapeutic success because exposure to the agent is increased by adjusting the dosing regimen or by its concentration at the site of infection

R resistant, a microorganism is categorized as resistant when there is a high likelihood of therapeutic failure, even when there is increased exposure

The *cfiA* gene was identified in 7/115 *B. fragilis* isolates (6.1%). The meropenem resistance in *B. fragilis* isolates, calculated according to the breakpoint reported in the version 12 and 13 EUCAST guidelines, was significantly higher than the one calculated following the v. 11 EUCAST guidelines (75% *vs*. 25%, *p* < 0.05). In *cfi*A-positive isolates, the MIC values were significantly higher for meropenem than for imipenem which proves that the use of meropenem better identifies carbapenem resistance in phenotypic testing. The meropenem MIC for c*fi*A-positive strains ranged from 1 to 32 mg/L and for *cfi*A-negative from 0.002 to 1 mg/L. The results of antibiotic susceptibility testing of all tested strains are included in the supplementary material.

**Fig. 1:**
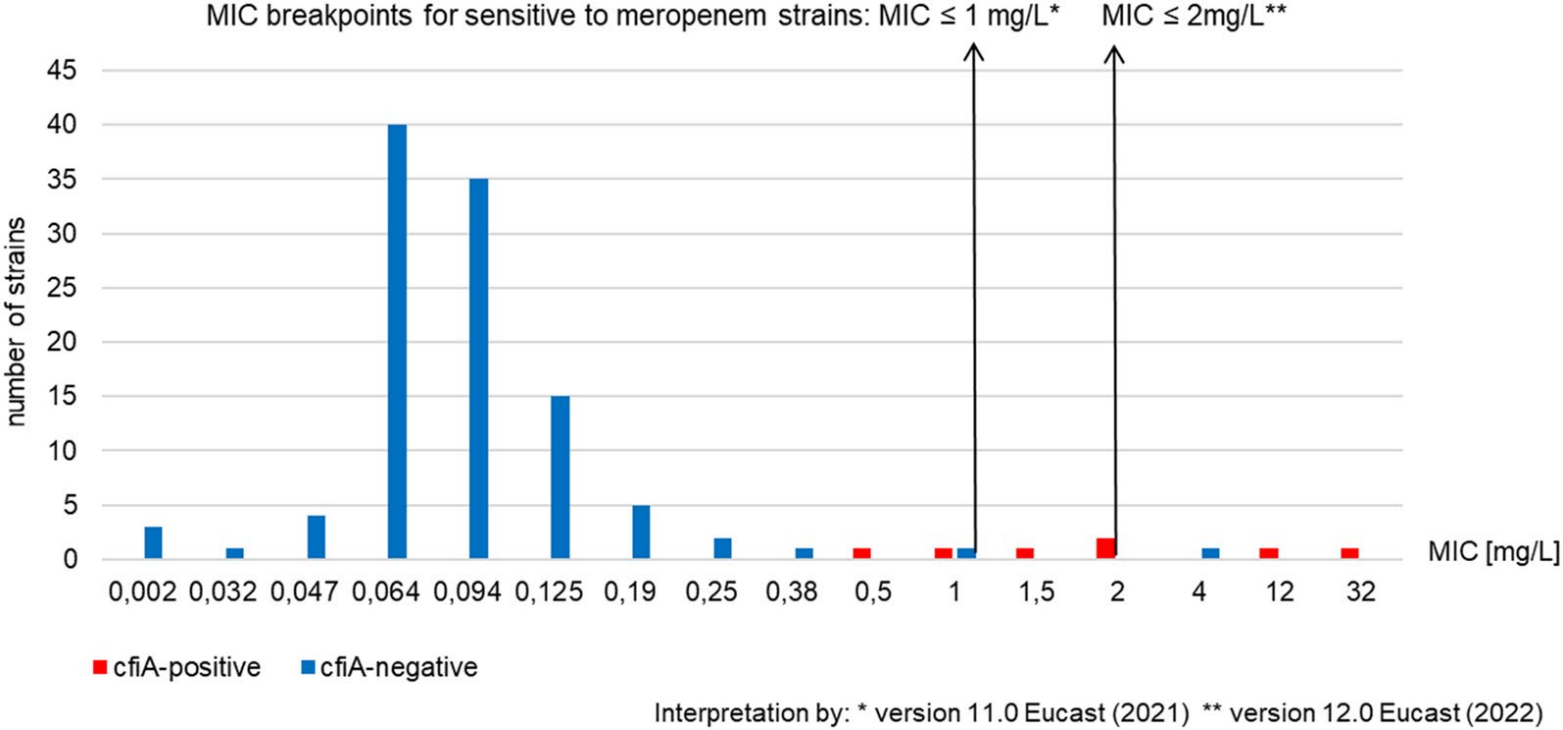
Correlation between meropenem MIC values and the presence of the *cfi*A gene in *B. fragilis* isolates [*n* = 115]

**Fig. 2:**
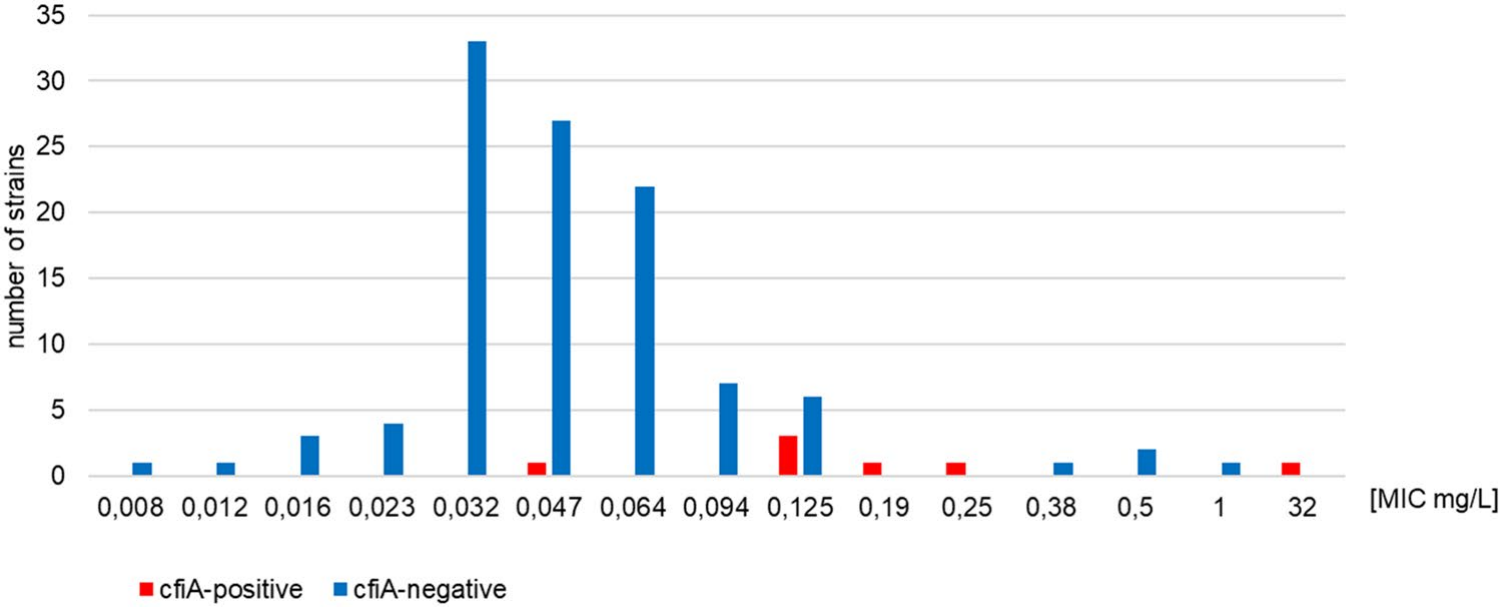
Correlation between imipenem MIC values and the presence of the *cfi*A gene in *B. fragilis* isolates [*n* = 115]

Figures 1 and 2 depict the correlation between imipenem and meropenem MIC values and the *cfi*A gene presence in *B. fragilis* isolates.

The presence of the *cfi*A gene weakly correlates with the MIC of imipenem and moderately correlates with the MIC of meropenem. The Pearson correlation coefficient equals 0.17 and 0.35 for imipenem and meropenem, respectively. The IS*1186* sequence was detected in one strain. The presence of the *cfi*A gene along with the IS*1186* was detected in a *B. fragilis* strain, which was susceptible to imipenem (MIC 0.125 mg/L) and resistant to meropenem (MIC 1.5 mg/L). No other insertion sequences (IS*1187*, IS*1188*, IS*942)* were detected in the screened strains.

The Carba NP test results were positive for all (seven) of the *cfi*A-positive strains, including two isolates susceptible to carbapenems based on their MIC values (0.5 and 1 mg/L). In two isolates that were phenotypically susceptible to meropenem (MIC 0.5 mg/L and 1 mg/L), *cfi*A gene and carbapenemase production were detected. In one *B. fragilis* strain (MIC of meropenem, 4 mg/L), the production of metallo-beta-lactamase was not detected (*cfi*A-negative), so other mechanisms produce carbapenem resistance.

## Discussion

*B. fragilis* is of particular clinical significance because of its numerous virulence factors such as capsular polysaccharides, iron acquisition, survival during the prolonged oxidative stress, quorum sensing, secretion of extracellular and histolytic enzymes, type VI secretion systems, and natural or acquired resistance to multiple antibiotics [38]. The plasticity of the *B. fragilis* genome allows it to incorporate virulence and related to antibiotic resistance determinants via horizontal gene transfer and to switch specific resistance genes on or off [39]. *B. fragilis* is responsible for purulent-septic infections, causes infections that result in high mortality rate, especially in the case of bacteremia [2, 11]. Enterotoxigenic *B. fragilis* (ETBF) strains are strongly associated with the occurrence of inflammatory bowel disease, colitis-associated colorectal cancer as well [40, 41, 42].

The carbapenems, including imipenem and meropenem, are active against anaerobic bacteria, but due to carbapenem resistance becoming increasingly more widespread, their use should be reserved for serious infections. There are reports, indicating that the frequency of carbapenem-resistant strains isolation is on an upward trend. It is necessary to detect resistance in isolates from patients and to monitor this phenomenon using sensitive and simple tests that could be used in the routine work of medical laboratories.

The β-lactam antibiotics resistance among *Bacteroides* spp. results from differing mechanisms. Of the greatest clinical and epidemiological significance is the production of different classes of β-lactamases, including the CfiA carbapenemase which hydrolyzes penicillins, cephalosporins, and carbapenems [17].

Carbapenem resistance may have other genetic causes, such as or reduced permeability of the outer membrane, over-expression of efflux pump genes (role in multiresistance, promoting MDR in *B. fragilis)*, or reduced affinity of penicillin binding proteins [1, 17, 39, 43].

This study determined the prevalence of *B. fragilis cfi*A-positive (Division II) isolates among strains isolated from infections of patients hospitalized in a large academic hospital in Warsaw, Poland. The influence of the presence of *cfi*A genes on phenotypic resistance to carbapenems was assessed, as well. The *cfiA* gene was identified in 6.1% *B. fragilis* isolates.

This subject has been widely studied by Jeverica et al. who screened a collection of *B. fragilis* isolates (623) using MALDI-TOF MS. Overall, 8.2% prevalence of Division II isolates (*cfi*A-positive) was detected in the two Slovenian tertiary care hospitals. A difference in proportion of *cfi*A-positive isolates between blood stream and non-blood stream specimens (14.9% vs. 7.6%; *p* = 0.081), was also revealed [17]. Ferløv-Schwensen and co-workers studied 444 *B. fragilis* group Danish clinical isolates and showed that from 1973–1980 to 2010–2015, the prevalence of antimicrobial resistance for meropenem rose from 0% to 2.5%. MALDI-TOF MS and real-time PCR identified 16 of 266 (6.0%) *B. fragilis* strains as Division II [44]. Overall, 7.8% (415 of 5300) *B. fragilis* clinical isolates studied by Cordovana et al*.* were found to belong to Division II, by the MALDI-TOF MS typing method, suspicious to harbor the *cfi*A gene in an active or inactive form. In all 70 *B. fragilis* strains typed by MALDI-TOF MS to belong to Division II PCR confirmed the presence of the *cfi*A gene. In seven *B. fragilis* isolates, IS elements upstream of the carbapenemase gene (IS*613*, IS*614B*, IS*942*, IS*1169*, or IS*1187*) were detected. All strains had a meropenem MIC ≥ 16 mg/L.

The Carba NP test detected carbapenemase activity in 6 of 29 (20.7%) Division II *B. fragilis* strains [45]. In a study typing 396 *B. fragilis* strains isolated from patients at Nagasaki University Hospital between 2006 and 2019, 8.3% harbored the *cfi*A gene. IS elements were found in seven *cfiA*-positive strains; IS*612*, IS*1187*, and IS*1188* were detected in each strains, and IS*612B* and IS*613* were detected each in two strain [46]. A review of the literature revealed that the rate of *B. fragilis* possessing the *cfiA* gene varies from 7.6% to 38.9% worldwide [14, 16, 17, 45, 46, 47]. The *cfi*A gene may be expressed at diverse levels, depending on the presence of IS upstream *cfi*A. In *B. fragilis*, IS*942,* IS*1186*, IS*1187*, IS*1188,* IS*612*, IS*613*, IS*614,* IS*615,* IS*616*, IS*4351,* have been related to *cfiA,* with varying promotion efficiency [48, 49]. In our study, IS*1186* was detected in only one strain. The presence of the *cfi*A gene along with the IS*1186* was detected in *B. fragilis* strain, which was susceptible to imipenem (MIC 0.125 mg/L) and resistant to meropenem (MIC 1.5 mg/L; Eucast 2022 and the latest). In the tested pool of isolates, there were *cfi*A-positive, but carbapenem susceptible isolates. It is known that *B. fragilis* with a *cfi*A gene can easily be converted to resistant genes by the effects of its upstream IS element, one-step mutation can allow the silent *cfi*A gene to be expressed [47, 48]. These results are in line with other European antimicrobial susceptibility studies [10, 17, 44, 48, 50, 51, 52, 53].

Phenotypic testing with the Carba NP test is a viable alternative for genetic technics for the presence of the *cfi*A gene in *Bacteroides* spp. The result of the Carba NP test together with the antibiogram allows to predict the effectiveness of therapy [28, 54].

In our study, the Carba NP test was positive for all *cfi*A-positive isolates including two strains phenotypically sensitive to meropenem with low MIC values (0.5 and 1 mg/L). In those two cases, low carbapenemase gene expression may occur. A clinical important issue is the possible conversion of meropenem-sensitive strains to resistant strains during therapy.

These results should prompt a discussion on whether carbapenem treatment is warranted when a strain of *B. fragilis* is *cfi*A-positive but phenotypically susceptible to meropenem. To date, there is little data on the clinical implications of such microbiological findings. It seems reasonable for clinicians to be cautious about treating infections with carbapenems, even if the isolate is phenotypically susceptible. Follow-up cultures monitoring the MIC of meropenem, to detect a potential increase in MIC values during treatment of the patient, are warranted [10, 17]. Javeriva and co-authors advise against amoxicillin or carbapenem therapy when Division II isolates are identified in their clinical centers [17]. This problem was also discussed by Hashimoto et al. who pointed out the importance of evaluating the use of meropenem as empirical therapy for *Bacteroides* sp. infections, considering the emergence of carbapenem resistance during treatment [55].

The need to monitor antibiotic susceptibility translates into biotechnological progress. Methods based on the mass spectrometry technique, not only precisely identify anaerobic bacteria, new versions of the software allow for the detection of resistance mechanisms and may be more sensitive and accurate than phenotypic methods [29, 45]. The sensitivity and specificity of the MALDI-TOF bacterial subtyping to detect *cfi*A in *B. fragilis* were 100.0 and 99.7%, respectively. Researchers find that the combination of MALDI-TOF MS and the Carba NP assay can be applied in diagnostic clinical laboratory for rapid identification of.

*B. fragilis* with IS element-activated *cfi*A gene [45, 46, 54]. It is cheaper and quicker than gene detection, which is a key factor in routine microbiological diagnostics.

There are several limitations to our study that need to be highlighted. First, it was a retrospective analysis that relied primarily on microbiological data and partial clinical data.

We lacked complete medical and treatment stories of the patients, specifically regarding their history of antibiotic therapy. This is particularly relevant concerning carbapenem therapy, which is a known selection factor for the expression of *cfi*A gene in Division II strains. Second, the study did not utilize more sensitive molecular methods such as Next Generation Sequencing (NGS) to explore carbapenem resistance in *cfi*A-negative *B. fragilis* isolates.

## Supplementary Information

Below is the link to the electronic supplementary material.

Supplementary file1 (DOCX 57 KB)

## Data Availability

The authors confirm that the data supporting the findings of this study are available within the article Phenotypic and genotypic identification of carbapenem resistance in Bacteroides fragilis clinical strains and its supplementary materials.
