# Supplementary material for: Phenotypic and genotypic identification of carbapenem resistance in Bacteroides fragilis clinical strains
Source: Med Microbiol Immunol. 2023 May 13;212(3):231–40. doi: 10.1007/s00430-023-00765-w (PMC10293361; doi:10.1007/s00430-023-00765-w)
Supplement: Supplementary file 1 — Supplementary file1 (DOCX 57 KB) [file 430_2023_765_MOESM1_ESM.docx]

**Supplementary material**

| **Nº** | **ID** | **MZ** | | **IP** | | **MP** | | ***XL** | | ***Pe** | | **CM** | | ***cfi*A/IS** |
| --- | --- | --- | --- | --- | --- | --- | --- | --- | --- | --- | --- | --- | --- | --- |
|  |  | MIC [mg/l] | | MIC [mg/l] | | MIC [mg/l]  (v.12 and v.13) | | MIC [mg/l] | | MIC [mg/l] | | MIC [mg/l] | |  |
| 1 | 2 | 0.38 | S | 0.047 | S | 0.094 | S | 0.25 | S | 12 | R | 0.75 | S | - |
| 2 | 3 | 0.25 | S | 0.032 | S | 0.094 | S | 0.19 | S | 8 | R | 0.5 | S | - |
| 3 | 8 | 0.094 | S | 0.064 | S | 0.064 | S | 0.19 | S | 48 | R | 0.047 | S | - |
| 4 | 12 | 0.125 | S | 0.25 | S | 12 | R (R) | 3 | S | 256 | R | 4 | S | + |
| 5 | 15 | 0.125 | S | 0.064 | S | 0.064 | S | 0.25 | S | 12 | R | 256 | R | - |
| 6 | 17 | 0.25 | S | 0.064 | S | 0.04 | S | 0.38 | S | 16 | R | 1 | S | - |
| 7 | 18 | 0.032 | S | 0.047 | S | 0.064 | S | 0.125 | S | 3 | R | 256 | R | - |
| 8 | 24 | 0.125 | S | 0.064 | S | 0.064 | S | 0.19 | S | 8 | R | 0.19 | S | - |
| 9 | 27 | 0.125 | S | 0.047 | S | 0.094 | S | 0.125 | S | 0.25 | S | 0.38 | S | - |
| 10 | 28 | 0.19 | S | 0.064 | S | 0.094 | S | 0.25 | S | 8 | R | 0.5 | S | - |
| 11 | 29 | 0.19 | S | 0.094 | S | 0.125 | S | 0.5 | S | 24 | R | 0.25 | S | - |
| 12 | 30 | 0.125 | S | 0.032 | S | 0.094 | S | 0.25 | S | 12 | R | 1.5 | S | - |
| 13 | 39 | 0.25 | S | 0.047 | S | 0.094 | S | 0.25 | S | 16 | R | 1 | S | - |
| 14 | 40 | 0.25 | S | 0.064 | S | 0.125 | S | 0.25 | S | 4 | R | >256 | R | - |
| 15 | 41 | 0.125 | S | 0.032 | S | 0.094 | S | 0.19 | S | 8 | R | 0.25 | S | - |
| 16 | 49 | 0.064 | S | 0.094 | S | 0.19 | S | 8 | I | >256 | R | 0.5 | S | - |
| 17 | 51 | 0.047 | S | 0.047 | S | 0.032 | S | 0.25 | S | 24 | R | 0.047 | S | - |
| 18 | 56 | 0.25 | S | 0.064 | S | 0.064 | S | 0.25 | S | 8 | R | 0.38 | S | - |
| 19 | 59 | 0.064 | S | 0.032 | S | 0.064 | S | 0.19 | S | 24 | R | 0.047 | S | - |
| 20 | 63 | 0.064 | S | 0.064 | S | 0.125 | S | 1 | S | 256 | R | 0.25 | S | - |
| 21 | 66 | 0.125 | S | 32 | R | 32 | R (R) | >256 | R | >256 | R | 2 | S | + |
| 22 | 68 | 0.064 | S | 0.047 | S | 0.094 | S | 0.25 | S | 12 | R | 0.25 | S | - |
| 23 | 70 | 0.19 | S | 0.047 | S | 0.094 | S | 0.38 | S | 8 | R | 1 | S | - |
| 24 | 71 | 0.19 | S | 0.032 | S | 0.064 | S | 0.38 | S | 16 | R | 0.5 | S | - |
| 25 | 73 | 0.125 | S | 0.064 | S | 0.094 | S | 0.19 | S | 6 | R | 0.064 | S | - |
| 26 | 76 | 0.032 | S | 0.047 | S | 0.5 | S (S) | 0.25 | S | 1 | R | 0.016 | S | + |
| 27 | 78 | 0.125 | S | 0.032 | S | 0.064 | S | 0.19 | S | 8 | R | 0.38 | S | - |
| 28 | 79 | 0.19 | S | 0.047 | S | 0.064 | S | 0.19 | S | 8 | R | 0.38 | S | - |
| 29 | 82 | 0.032 | S | 0.125 | S | 1 | S (S) | 1 | S | 2 | R | 0.75 | S | + |
| 30 | 84 | 0.125 | S | 0.064 | S | 0.064 | S | 0.5 | S | 32 | R | 1.5 | S | - |
| 31 | 85 | 0.25 | S | 0.064 | S | 0.064 | S | 0.5 | S | 12 | R | 0.5 | S | - |
| 32 | 86 | 0.25 | S | 0.047 | S | 0.094 | S | 0.38 | S | 8 | R | >256 | R | - |
| 33 | 87 | 0.25 | S | 0.125 | S | 1.5 | S (R) | 1.5 | S | 4 | R | 3 | S | +/ IS1186 |
| 34 | 88 | 0.094 | S | 0.19 | S | 2 | S (R) | 1 | S | 6 | R | 0.75 | S | + |
| 35 | 96 | 0.38 | S | 0.064 | S | 0.094 | S | 1 | S | 256 | R | 1.5 | S | - |
| 36 | 97 | 0.25 | S | 0.032 | S | 0.064 | S | 0.25 | S | 8 | R | 0.25 | S | - |
| 37 | 98 | 0.064 | S | 0.047 | S | 0.094 | S | 0.75 | S | 256 | R | 0.125 | S | - |
| 38 | 101 | 0.125 | S | 0.032 | S | 0.094 | S | 0.19 | S | 4 | R | 3 | S | - |
| 39 | 103 | 0.125 | S | 0.047 | S | 0.064 | S | 0.38 | S | 4 | R | 0.064 | S | - |
| 40 | 104 | 0.094 | S | 0.094 | S | 0.125 | S | 8 | I | 256 | R | 256 | R | - |
| 41 | 114 | 0.19 | S | 0.032 | S | 0.125 | S | 0.25 | S | 12 | R | 0.5 | S | - |
| 42 | 115 | 0.25 | S | 0.125 | S | 2 | S (R) | 1 | S | 4 | R | 0.125 | S | + |
| 43 | 116 | 0.125 | S | 0.064 | S | 0.094 | S | 0.25 | S | 16 | R | 256 | R | - |
| 44 | 120 | 0.19 | S | 0.064 | S | 0.094 | S | 1 | S | 256 | R | 256 | R | - |
| 45 | 121 | 0.19 | S | 0.047 | S | 0.094 | S | 0.25 | S | 8 | R | 1 | S | - |
| 46 | 123 | 0.38 | S | 0.064 | S | 0.064 | S | 0.38 | S | 16 | R | 0.19 | S | - |
| 47 | 124 | 0.094 | S | 0.032 | S | 0.064 | S | 0.25 | S | 8 | R | 0.094 | S | - |
| 48 | 125 | 0.38 | S | 0.047 | S | 0.064 | S | 0.38 | S | 16 | R | 0.38 | S | - |
| 49 | 129 | 0.25 | S | 0.064 | S | 0.064 | S | 0.25 | S | 24 | R | 0.064 | S | - |
| 50 | 132 | 0.19 | S | 0.047 | S | 0.094 | S | 0.25 | S | 3 | R | 0.38 | S | - |
| 51 | 133 | 0.016 | S | 0.032 | S | 0.125 | S | 0.016 | S | 3 | R | 0.047 | S | - |
| 52 | 137 | 0.19 | S | 0.032 | S | 0.094 | S | 0.25 | S | 4 | R | 0.5 | S | - |
| 53 | 141 | 0.38 | S | 0.047 | S | 0.094 | S | 0.25 | S | 12 | R | 0.5 | S | - |
| 54 | 144 | 0.25 | S | 0.064 | S | 0.064 | S | 0.25 | S | 4 | R | 0.125 | S | - |
| 55 | 146 | 0.38 | S | 0.047 | S | 0.094 | S | 0.25 | S | 8 | R | 1 | S | - |
| 56 | 148 | 0.25 | S | 0.064 | S | 0.064 | S | 0.38 | S | 12 | R | 1.5 | S | - |
| 57 | 151 | 0.125 | S | 0.047 | S | 0.064 | S | 0.19 | S | 3 | R | 0.75 | S | - |
| 58 | 154 | 0.125 | S | 0.5 | S | 1 | S | 1.5 | S | 256 | R | 0.38 | S | - |
| 59 | 157 | 0.25 | S | 0.5 | S | 0.094 | S | 0.25 | S | 6 | R | 1.5 | S | - |
| 60 | 161 | 0.25 | S | 1 | S | 0.38 | S | 12 | R | 256 | R | 0.125 | S | - |
| 61 | 162 | 0.19 | S | 0.125 | S | 0.125 | S | 0.75 | S | 256 | R | 0.19 | S | - |
| 62 | 163 | 0.38 | S | 0.047 | S | 0.094 | S | 0.5 | S | 4 | R | 1 | S | - |
| 63 | 166 | 0.25 | S | 0.125 | S | 0.125 | S | 0.75 | S | 256 | R | 0.047 | S | - |
| 64 | 167 | 0.19 | S | 0.125 | S | 0.125 | S | 1 | S | 256 | R | 0.5 | S | - |
| 65 | 180 | 0.047 | S | 0.047 | S | 0.094 | S | 0.125 | S | 3 | R | 0.12 | S | - |
| 66 | 182 | 0.19 | S | 0.047 | S | 0.064 | S | 0.19 | S | 6 | R | 0.064 | S | - |
| 67 | 189 | 0.38 | S | 0.032 | S | 0.094 | S | 0.25 | S | 4 | R | 0.125 | S | - |
| 68 | 191 | 0.19 | S | 0.094 | S | 0.064 | S | 0.25 | S | 12 | R | 0.38 | S | - |
| 69 | 196 | 0.19 | S | 0.094 | S | 0.125 | S | 0.75 | S | 256 | R | 256 | R | - |
| 70 | 197 | 0.25 | S | 0.023 | S | 0.064 | S | 0.125 | S | 4 | R | 0.38 | S | - |
| 71 | 201 | 0.094 | S | 0.032 | S | 0.064 | S | 0.19 | S | 4 | R | 256 | R | - |
| 72 | 206 | 0.19 | S | 0.032 | S | 0.064 | S | 0.19 | S | 8 | R | 0.064 | S | - |
| 73 | 208 | 0.25 | S | 0.047 | S | <0.002 | R | 0.19 | S | 12 | R | >256 | R | - |
| 74 | 209 | 0.25 | S | 0.032 | S | 0.064 | S | 0.19 | S | 12 | R | >256 | R | - |
| 75 | 212 | 0.064 | S | 0.032 | S | 0.064 | S | 0.25 | S | 1.5 | R | 0.032 | S | - |
| 76 | 214 | 0.047 | S | 0.008 | S | <0.002 | R | 0.016 | S | 0.016 | S | 0.094 | S | - |
| 77 | 221 | 0.023 | S | 0.064 | S | 4 | I (R) | 1.5 | S | 12 | R | 0.047 | S | - |
| 78 | 223 | 0.094 | S | 0.094 | S | 0.125 | S | 1 | S | 256 | R | 0.064 | S | - |
| 79 | 224 | 0.016 | S | 0.047 | S | <0.002 | R | 0.19 | S | 8 | R | 0.38 | S | - |
| 80 | 225 | 0.125 | S | 0.032 | S | 0.064 | S | 0.125 | S | 12 | R | 0.38 | S | - |
| 81 | 228 | 0.19 | S | 0.047 | S | 0.25 | S | 0.25 | S | 8 | R | 1 | S | - |
| 82 | 230 | 0.094 | S | 0.032 | S | 0.047 | S | 0.047 | S | 6 | R | 0.047 | S | - |
| 83 | 232 | 0.064 | S | 0.016 | S | 0.064 | S | 0.125 | S | 6 | R | 0.016 | S | - |
| 84 | 233 | 0.064 | S | 0.032 | S | 0.094 | S | 0.125 | S | 6 | R | 0.5 | S | - |
| 85 | 238 | 0.19 | S | 0.047 | S | 0.047 | S | 0.19 | S | 8 | R | 1 | S | - |
| 86 | 241 | 0.25 | S | 0.016 | S | 0.064 | S | 0.047 | S | 0.19 | S | 0.16 | S | - |
| 87 | 244 | 0.125 | S | 0.032 | S | 0.094 | S | 0.125 | S | 4 | R | 0.125 | S | - |
| 88 | 254 | 0.094 | S | 0.012 | S | 0.047 | S | 0.094 | S | 0.064 | S | 0.016 | S | - |
| 89 | 260 | 0.125 | S | 0.032 | S | 0.094 | S | 0.19 | S | 4 | R | 0.5 | S | - |
| 90 | 261 | 0.25 | S | 0.032 | S | 0.064 | S | 0.25 | S | 6 | R | 256 | R | - |
| 91 | 262 | 0.25 | S | 0.023 | S | 0.094 | S | 0.25 | S | 8 | R | 256 | R | - |
| 92 | 264 | 0.25 | S | 0.064 | S | 0.125 | S | 0.5 | S | 256 | R | 256 | R | - |
| 93 | 268 | 0.094 | S | 0.032 | S | 0.064 | S | 0.125 | S | 6 | R | 256 | R | - |
| 94 | 277 | 0.125 | S | 0.023 | S | 0.064 | S | 0.25 | S | 4 | R | 0.032 | S | - |
| 95 | 278 | 0.19 | S | 0.023 | S | 0.125 | S | 0.125 | S | 1 | R | 1.5 | S | - |
| 96 | 284 | 0.125 | S | 0.016 | S | 0.064 | S | 0.094 | S | 2 | R | 256 | R | - |
| 97 | 286 | 0.25 | S | 0.032 | S | 0.064 | S | 0.19 | S | 6 | R | 256 | R | - |
| 98 | 288 | 0.032 | S | 0.38 | S | 0.19 | S | 0.5 | S | 256 | R | 0.032 | S | - |
| 99 | 291 | 0.094 | S | 0.125 | S | 0.19 | S | 3 | S | 256 | R | 0.094 | S | - |
| 100 | 298 | 0.38 | S | 0.032 | S | 0.094 | S | 0.25 | S | 2 | R | 256 | R | - |
| 101 | 299 | 0.19 | S | 0.032 | S | 0.064 | S | 0.19 | S | 1.5 | R | 0.094 | S | - |
| 102 | 301 | 0.25 | S | 0.047 | S | 0.094 | S | 0.38 | S | 16 | R | 0.5 | S | - |
| 103 | 305 | 0.19 | S | 0.047 | S | 0.064 | S | 0.19 | S | 2 | R | 0.016 | S | - |
| 104 | 306 | 0.38 | S | 0.032 | S | 0.064 | S | 0.125 | S | 0.25 | S | 0.064 | S | - |
| 105 | 307 | 0.25 | S | 0.032 | S | 0.094 | S | 0.125 | S | 2 | R | 0.25 | S | - |
| 106 | 407 | 0.25 | S | 0.064 | S | 0.19 | S | 0.38 | S | 24 | R | 0.094 | S | - |
| 107 | 411 | 0.19 | S | 0.125 | S | 0.125 | S | 1 | S | 256 | R | 256 | R | - |
| 108 | 413 | 0.025 | S | 0.064 | S | 0.094 | S | 0.25 | S | 2 | R | 0.25 | S | - |
| 109 | 414 | 0.19 | S | 0.032 | S | 0.125 | S | 0.125 | S | 1 | R | 0.5 | S | - |
| 110 | 419 | 0.094 | S | 0.032 | S | 0.064 | S | 0.125 | S | 4 | R | 0.5 | S | - |
| 111 | 420 | 0.25 | S | 0.047 | S | 0.064 | S | 0.19 | S | 3 | R | 0.75 | S | - |
| 112 | 422 | 0.25 | S | 0.032 | S | 0.094 | S | 0.19 | S | 6 | R | 256 | R | - |
| 113 | 424 | 0.25 | S | 0.094 | S | 0.25 | S | 1.5 | S | 256 | R | 1.5 | S | - |
| 114 | 427 | 0.19 | S | 0.032 | S | 0.094 | S | 0.125 | S | 2 | R | 0.25 | S | - |
| 115 | 429 | 0.094 | S | 0.125 | S | 0.19 | S | 0.75 | S | 256 | R | 256 | R | - |

**Table 1.** Characterization of *Bacteroides fragilis* strains isolated from clinical specimens taken from patients hospitalized in academic hospital in Warsaw, Poland, the Medical University of Warsaw during a 5‐year period (2013‐2017).

**Legend:**
MZ; metronidazole, IP; imipenem, MP; meropenem, CM; clindamycin, XL; amoxicillin/clavulanic acid,
Pe; benzylpenicillin,
S; susceptible, R; resistant, I; intermediate (susceptible, increased exposure).

**Comments:**MIC breakpoints interpretation for **MZ**, **IP** and **CM** according to EUCAST recommendation version 12 (year 2022) in line with the version 13 (2023).
MIC breakpoints interpretation for **MP** according to EUCAST recommendation version 11 (year 2021); in brackets interpretation according to v. 12 (year 2022) complies with v.13 (year 2023). Changes in the recommendation have affected susceptibility interpretation to meropenem.
MIC breakpoints interpretation for **XL** and **Pe** according to EUCAST recommendation version 11 (year 2021). Susceptibility to ampicillin, amoxicillin and piperacillin can be predicted by susceptibility testing to benzylpenicillin.
EUCAST announced that amoxicillin/clavulanic acid breakpoints will be developed during 2022 (no published to date).

* Presently, benzylpenicillin is not recommend for *Bacteroides* spp. infection therapy, susceptibility testing not longer recommended. Most *Bacteroides* isolates are likely β-lactamase producers or are resistant to penicillin by other mechanisms. Because alternative mechanisms of resistance to β-lactams exist, a negative test (using a chromogenic assay as a nitrocefin disk assay or an S1 chromogenic disk) does not assure susceptibility to penicillin.
